# Supplementary material for: Establishment of an early diagnosis model of colon cancerous bowel obstruction based on 1H NMR
Source: PLoS One. 2022 Aug 16;17(8):e0266730. doi: 10.1371/journal.pone.0266730 (PMC9380946; doi:10.1371/journal.pone.0266730)
Supplement: S1 Data — (DOCX) [file pone.0266730.s001.docx]

This is the raw metabolomics data from the article Establishment of an early detection model for colon malignant bowel obstruction using 1H NMR, where the file Baseline data20211218 is located (1). The data file 52X15metaboanalyse2.csv contains a data table describing the relationship between the spectral data and the samples after filtering.

The data above have been evaluated by the hospital's ethical committee and do not include any personally identifiable information about patients.

the raw data of the desensitized papers can be downloaded at https://github.com/dcpengjin/metabolomics_data
