# Supplementary material for: Establishment of an early diagnosis model of colon cancerous bowel obstruction based on 1H NMR
Source: PLoS One. 2022 Aug 16;17(8):e0266730. doi: 10.1371/journal.pone.0266730 (PMC9380946; doi:10.1371/journal.pone.0266730)
Supplement: Attachment — Submitted filename: Response to reviewer.docx [file pone.0266730.s002.docx]

**Response to academic editor**：

We really appreciate you for your carefulness and conscientiousness. Your suggestions are really valuable and helpful for revising and improving our paper. According to your suggestions, we have made the following revisions on this manuscript:

**1**. Please ensure that your manuscript meets PLOS ONE's style requirements, including those for file naming. The PLOS ONE style templates can be found athttps://journals.plos.org/plosone/s/file?id=wjVg/PLOSOne_formatting_sample_main_body.pdf and https://journals.plos.org/plosone/s/file?id=ba62/PLOSOne_formatting_sample_title_authors_affiliations.pdf.

**Response**：Thank you for your detailed comments. We have revised the manuscript according to the PLOSOne guideline thoroughly, which are highlighted in red in the revised manuscript.

**2**. Please ensure that you include a title page within your main document. We do appreciate that you have a title page document uploaded as a separate file, however, as per our author guidelines (http://journals.plos.org/plosone/s/submission-guidelines#loc-title-page) we do require this to be part of the manuscript file itself and not uploaded separately.

Could you therefore please include the title page into the beginning of your manuscript file itself, listing all authors and affiliations.

**Response**：Thank you very much for your advice. The title page have been added into the beginning which are highlighted in red in the revised manuscript. (page 1, lines5-12)

**3**. We suggest you thoroughly copyedit your manuscript for language usage, spelling, and grammar.

A copy of your manuscript showing your changes by either highlighting them or using track changes (uploaded as a *supporting information* file)

A clean copy of the edited manuscript (uploaded as the new *manuscript* file).

**Response**：Thank you for your detailed comments. We have thoroughly copyedited the manuscript for language usage, spelling, and grammar. The amendments are highlighted in red in the revised manuscript.( page 1, lines1,30,38; page3, line42; page5, lines16,18-20; page 6, lines9,12,25-26; page 7, lines11-12; page 9, lines6-7.)

**4**. We note that the grant information you provided in the ‘Funding Information’ and ‘Financial Disclosure’ sections do not match.

When you resubmit, please ensure that you provide the correct grant numbers for the awards you received for your study in the ‘Funding Information’ section.

**Response**：Thank you for your detailed comments. The correct grant numbers is 2018YSKY0017-9( Basic scientific research business expenses of Science ＆ Technology Department of Sichuan Province) , which is also corrected in the ‘Funding Information’ section.

**5**. Thank you for stating the following in the Acknowledgments Section of your manuscript:“The present study was supported by Basic scientific research business expenses of public welfare scientific research institutes of Sichuan Province, China (grant no. 30504010428).”

We note that you have provided funding information that is not currently declared in your Funding Statement. However, funding information should not appear in the Acknowledgments section or other areas of your manuscript. We will only publish funding information present in the Funding Statement section of the online submission form.

Please remove any funding-related text from the manuscript and let us know how you would like to update your Funding Statement. Currently, your Funding Statement reads as follows:

“No, The funders had no role in study design, data collection and analysis, decision to publish, or preparation of the manuscript”. Please include your amended statements within your cover letter; we will change the online submission form on your behalf.

**Response**：Thank you for your positive comments. We have removed funding-related text from the manuscript. The study was supported by Basic scientific research business expenses of Science ＆ Technology Department of Sichuan Province (grant no.2018YSKY0017-9), and the funders had no role in study design, data collection and analysis, decision to publish, or preparation of the manuscript. Kexun Li and Daolin Long who were studying for a master's degree got ￥12,000 as Labor wage from this foundation, respectively. Mean while, the other authors of this manuscript got no salary from the funder.

**6**. Thank you for stating the following in your Competing Interests section: “NO authors have competing interests”

Please complete your Competing Interests on the online submission form to state any Competing Interests. If you have no competing interests, please state ""The authors have declared that no competing interests exist."", as detailed online in our guide for authors at http://journals.plos.org/plosone/s/submit-now

This information should be included in your cover letter; we will change the online submission form on your behalf.

**Response**：Thank you for your detailed comments. The authors have declared that no competing interests exist, which are highlighted in red in the revised manuscript. (page 9, lines 29-30)

**7**. Please include your full ethics statement in the ‘Methods’ section of your manuscript file. In your statement, please include the full name of the IRB or ethics committee who approved or waived your study, as well as whether or not you obtained informed written or verbal consent. If consent was waived for your study, please include this information in your statement as well.

**Response**： The full ethics statement has been included in the ‘Methods’ section of the manuscript file, which is highlighted in red in the revised manuscript.( page 2, lines 19)

**Response to reviewer #1:**

We really appreciate you for your carefulness and conscientiousness. Your suggestions are really meaningful and useful for revising and improving this paper. According to your suggestions, we have made the following revisions on this manuscript:

**1**. In this manuscript 1H NMR coupled with machine learning is used to build a model for the differential diagnosis of acute colon cancerous bowel obstruction (CBO) vs. adhesive bowel obstruction (ABO) in serum. Although the entire spectrum is proposed to function as “biomarker”, then the authors concentrate the discussion on six metabolites.

It would be important to know what would be the prediction accuracy, sensitivity and specificity of a model based on these metabolites rather than on the whole binned spectrum, to evaluate the contribution of the other molecules.

**Response**：We appreciate the reviewers' feedback. Regarding the accuracy, sensitivity, and specificity of the model composed of six metabolites in comparison to the entire classification spectrum for predicting CBO, we searched the HMDB for 1470 possible metabolites after obtaining 35 segments of chemical shifts with VIP values greater than 1 via PCA and PLS-DA analysis of the spectral matrix, of which 388 possible endogenous metabolites were detected in blood. According to the Jaccard value, the top 30 metabolites were selected. By comparing standard spectra with HMDB (1H NMR Spectrum [1D, 600 MHz, D2O, anticipated]), it was shown that 15 metabolites had the lowest peaks at chemical shifts that overlapped with the above 35 segments. Mnova software was used to determine the peak intensities of these 15 shifts separately, and a t-test revealed that the peak intensities of six shifts were substantially different. Six chemical shifts correspond to six metabolites, and the integrals of the lowest peak intensity are notably different	S2 Table. We have revised the article in light of the reviewers' comments. (page 5, lines 2-28)

**2**. The NMR analysis was limited to the acquisition of CPMG spectra, thus excluding all lipoproteins components, which would help defining metabolic alterations. It is a common practice in 1H NMR metabolomics to use at least NOESY and CPMG experiments.

**Response**：We appreciate your kind words. Due to financial and time constraints, we have only completed the 1H NMR test at this stage. We will use GC-MS or LC-MS for the mass spectrometry test verification of the above substances in the next stage. We have also revised the article in light of the reviewers' comments. (page 8, line 41)

**3**. The serum handling procedure are largely divergent with respect to ISO standards (ISO 23118:2021 Molecular in vitro diagnostic examinations — Specifications for pre-examination processes in metabolomics in urine, venous blood serum and plasma), which could in principle affect the outcome of the downstream metabolomics analysis. Possibly the adopted procedure does not influence the comparison between the two groups of patients enrolled in the same study, but it might affect the general applicability of the model.

**Response**： Regarding the specimen handling process, we made an error expression: Because of the particularity of emergency patients (more admitted at night), our plan was to draw whole blood from the peripheral veins of all participants within 2 hours after admission, and used vacuum blood collection tubes to collect 3ml of whole blood for each participant (vacuum tube with blue cap without addition, 10.25mm×64mm, batch number 363095, American BD company), immediately put it in a refrigerator at -20℃, and centrifuge at 3000r/min for 15min within 48h, and took the supernatant , Transferred to EP tube, -80 ℃ refrigerator for refrigeration. But the actual operation was to draw 3ml of whole blood from the peripheral vein within 2 hours after admission into the tubes (Blue cap with sodium citrate, 10.25mm×64mm, batch number 363095, US BD company), centrifuged inside at 16000r/min for 15min in 30 minutes, took the supernatant, transferred to EP tube, and refrigerate at -80℃. After the specimens were collected, they were transferred to the laboratory to thaw at room temperature and transferred to a 5mm Wilmad NMR tube for on-board testing and analysis. Although the thawing temperature is different from that specified in ISO 23118:2021 and requires extended time detection, we believe that, while the serum handling procedures are different, the differences determined by the method based on the overall molecular magnetic resonance hydrogen spectroscopy profile are similar. That is, our method, PLS-DA, PCA, relies heavily on linear transformations to identify the components with the highest variation among the different grouped samples, and so is mostly unaffected by the aforementioned criteria. We have also revised the article in light of the reviewers' comments. (page 3, lines 22-29, 32-33; )

**4**. It would be also important to know what has been administered to the patients in the time interval between admission and blood collection (e.g. Ringer acetate solutions or contrast agents for imaging, and not just “drugs” affect the NMR metabolic profiles

**Response**：Thank you very much for your advice. Within 48 hours, none of our patients had medical procedures (e.g., oral or intravenous administration, gastrointestinal decompression, enemas), with the exception of fasting and water fasting for >24 hours, which we included in our exclusion criteria. No patient received imaging examination using contrast agents, and no patient got intravenous or oral rehydration prior to specimen collection. We have already revised the article in light of the reviewers' comments. (page 2, lines 38,44; page 3, lines 1-3 )

**5**. Formal aspects:
Missing information: the data availability statement does not describe where the data can be found.

**Response**：Thank you for your constructive comments. In accordance with the principles of medical ethics, the raw data of the desensitized papers can be downloaded at https://github.com/dcpengjin/metabolomics_data.git

**6**. Spelling:

Throughout the text: 1H NMR

Page 10, … 5 seconds

Page 12 (Discussion), … between the two groups.

**Response**： We appreciate the reviewers' helpful and constructive suggestions for grammar and spelling changes. All errors mentioned above have been corrected. We have already revised the article in light of the reviewers' comments. (page 1, lines 2,15; page 3, lines 41-42; page 6, lines 9,12,26)
