# Supplementary material for: Establishment of an early diagnosis model of colon cancerous bowel obstruction based on 1H NMR
Source: PLoS One. 2022 Aug 16;17(8):e0266730. doi: 10.1371/journal.pone.0266730 (PMC9380946; doi:10.1371/journal.pone.0266730)
Supplement: Attachment — Submitted filename: Response to reviewer.docx [file pone.0266730.s003.docx]

**Response to academic editor**：

Your suggestions are really valuable and helpful for revising and improving our paper. According to your suggestions, we have made the following revisions on this manuscript:

**1**. Please review your reference list to ensure that it is complete and correct. If you have cited papers that have been retracted, please include the rationale for doing so in the manuscript text, or remove these references and replace them with relevant current references. Any changes to the reference list should be mentioned in the rebuttal letter that accompanies your revised manuscript. If you need to cite a retracted article, indicate the article’s retracted status in the References list and also include a citation and full reference for the retraction notice.

**Response**：Thank you for your detailed comments. We have reviewed our reference list thoroughly to ensure that it is complete and correct. we did not find any records about being retracted of these papers that we cited in the manuscript. In this revision, we cited another three papers.The amendments are highlighted in red in the revised manuscript.( lines195,216,339,424-427,498-500.)

**Response to reviewer #1:**

Your suggestions are really meaningful and useful for revising and improving this paper. According to your suggestions, we have made the following revisions on this manuscript:

**1**. The question was very simple: what is the difference in prediction accuracy, sensitivity and specificity if the authors use the entire binned spectrum? This procedure does not require any assignment (for details see for example https://doi.org/10.1016/j.trac.2018.10.036). I invite the authors to test this approach.

**Response**： The papers you recommended are very valuable to us. We performed ROC analysis on the 6 metabolites which have significant differences between the two groups and the full-spectrum prediction model. According to the AUC value, the prediction accuracy of the full-spectrum model was higher. For the verified source code see: https://github.com/dcpengjin/metabolomics_data.git. We also have revised the article in light of the reviewers' comments. (lines 192-195)

**2**. I was suggesting to use 1H NMR NOESY spectra to derive information on the lipoprotein components (see Bruker IVDR tool; https://doi.org/10.1021/acs.analchem.8b02412), not to perform MS analyses.

**Response**：Thank you very much for your professional advice and reminders. Our original purpose was to observe small molecules through a relatively faster 1H-NMR test. Due to the economic and time constraints, this study did not carry out NOESY test, so it was flawed in identifying lipoproteins, and the analysis of macromolecular proteins will be further discussed in future experiments. We have also revised the article in light of the reviewers' comments. (line 337-341)
